# Development and preliminary validation of a PROS scale for Chinese bladder cancer patients with abdominal stoma

**DOI:** 10.1038/s41598-024-52624-0

**Published:** 2024-01-25

**Authors:** Jingya Lu, Hui Hong, Zhufeng Xiong, Yonghui Zhang, Fanyan Zeng, Zhiqin Xie, Mengjia Yu, Xiaohan Liu, Huiting Li, Daming Xian, Junjie Shen

**Affiliations:** 1 Department of Nursing, The 1st Affiliated Hospital of Nanchang University, Nanchang, Jiangxi China; 2 School of Nursing, Jiangxi Medical College, Nanchang University, Nanchang, Jiangxi China; 3 Intensive Care Unit, Department of Infection, The 1st Affiliated Hospital of Nanchang University, Nanchang, Jiangxi China; 4 School of Public Health, Jiangxi Medical College, Nanchang University, Nanchang, Jiangxi China; 5 Jiangxi Medical Center for Critical Public Health Events, The First Affiliated Hospital, Jiangxi Medical College, Nanchang University, Nanchang, Jiangxi China; 6 The Second Affiliated Hospital of Nanchang University, Nanchang, Jiangxi China; 7 First Affiliated Hospital of Dalian Medical University, Dalian, Liaoning China; 8 College of Mathematics and Computer Science, Nanchang University, Nanchang, Jiangxi China; 9 School of Software, Nanchang University, Nanchang, Jiangxi China

**Keywords:** Oncology, Urology

## Abstract

Bladder cancer is a common malignant tumor, and patients who have undergone radical cystectomy and urinary diversion require a lifelong abdominal stoma. This greatly affects their physiological, psychological, and social well-being. However, there is currently a lack of a self-assessment outcome scale specifically designed for bladder cancer patients with abdominal stomas. Therefore, we developed and validated a self-assessment outcome scale (PROS-BCAS) for Chinese bladder cancer patients with abdominal stomas. The scale was initially developed through literature research and expert consultation, and it comprised four dimensions: physiological, psychological, social, and treatment, with a total of 66 items. After item analysis, 44 items were retained. We collected scale data from 382 patients to examine its validity and reliability. The results showed that the PROS-BCAS scale had good content validity (S-CVI/Ave = 0.992), construct validity (KMO > 0.6), and discriminant validity (correlation coefficient 0.404–0.870). The Cronbach's alpha coefficients (0.801–0.954), test–retest reliability (0.778–0.956), and split-half reliability (0.896–0.977) all demonstrated good internal consistency for each dimension and the overall scale. The study demonstrated that the PROS-BCAS scale is a reliable and valid tool for accurately assessing the health-related quality of life of bladder cancer patients with abdominal stomas, providing reference for developing individualized clinical care plans.

## Introduction

Bladder Cancer (BCa) is one of the most common malignancies worldwide^1^. According to the latest statistics from GLOBOCAN^2^, the number of bladder cancer patients worldwide reached 573,000 in 2020, making it the 12th leading cause of malignant tumor incidence. This statistical data reveals the severity of bladder cancer as an important public health issue^3^. Especially in Europe, North America, and China, the incidence of bladder cancer is particularly significant in elderly males^4^, posing new challenges for public health policy formulation and allocation of medical resources in our country^3,5,6^. Particularly noteworthy is that muscle-invasive bladder cancer (MIBC) accounts for 25% of all bladder cancer cases and has a higher risk of recurrence and metastasis^7^. Patients with advanced disease often require Radical Cystectomy (RC) and Urinary Diversion (UD)^8^, which include Orthotopic Bladder Substitution (OBS), Ileal Conduit (IC), and Cutaneous Ureterostomy (CU)^9^. After undergoing IC or CU, patients need to wear urinary bags for life, which has a significant impact on their quality of life^10,11^.

Existing studies have already shown that the quality of life of bladder cancer patients with abdominal wall stomas declines in multiple aspects such as physiology, psychology, role, and social function^12–14^. The alteration of physiological urinary pathway due to surgery and the issues that may arise from long-term wearing of urinary bags, such as odor and skin irritation, can have negative effects on patients' self-image and social ability^15,16^. In addition, factors such as the need for lifelong placement and regular replacement of ureteral stents, the consumption of stoma-related products, and the management of complications further increase the economic burden on patients^17,18^, thereby further affecting their physiological, psychological, and social functions^19,20^. However, the traditional evaluation system focuses primarily on treatment outcomes and often overlooks these potential impacts^21,22^. Although we have begun to recognize this problem, there is still a lack of a professional scale that can quantify these effects.

To address this issue, we have decided to adopt the assessment of Patient-Reported Outcomes (PROs) and focus on Health-Related Quality of Life (HRQOL)^23^. Patient-Reported Outcomes (PROs) is a broad concept that represents patients' subjective reports and evaluations of their disease and treatment outcomes. This includes patients' perceptions of their symptoms, functional status, overall health, and treatment satisfaction^24,25^. In this framework, we define the PROS-BCAS (Patient-Reported Outcomes Scale for Bladder Cancer Abdominal Stoma) as the subjective report and evaluation of bladder cancer patients' experience, including symptoms, functional status, overall health, and treatment satisfaction. Health-Related Quality of Life (HRQOL) is an important aspect of PROs that mainly focuses on how disease and treatment affect patients' daily quality of life^26^. From the perspective of the "biopsychosocial" model of medicine and the patient-centered approach, this assessment method is in line with the concept of modern clinical medicine^27^. The application scope of PROs, which has been recognized by institutions such as the U.S. Food and Drug Administration (FDA), has gradually expanded to various fields, including clinical medical research, drug clinical trials, healthcare quality assessment, and health policy formulation^28^. More importantly, the use of PROs can significantly improve patient prognosis in clinical care, including symptom relief, treatment adherence, and patient satisfaction^29,30^.

Therefore, the objective of this study is to develop a self-report outcomes scale specifically for patients with bladder cancer abdominal stomas in the context of China. Considering the unique cultural and social environment in China, as well as the special needs of Chinese bladder cancer patients, we believe it is necessary to develop a tool specifically for this population. Although there are already some available bladder cancer patient scales^31–33^, there is still a lack of a self-report outcomes scale specifically for patients with bladder cancer abdominal stomas. We hope that this tool can provide important references on the health status of these patients, optimize clinical outcomes, and determine personalized best care practices.

## Methods

### Construction of questionnaire item pool

We conducted a literature search in the Chinese databases CNKI and CBM, as well as the databases Wanfang, VIP, PubMed, Embase, Web of Science, and CINAHL for relevant studies. The search keywords and subject terms included bladder tumor, bladder cancer, urinary diversion, urinary stoma, cutaneous ureterostomy, bladder cancer with abdominal stoma, radical cystectomy, total cystectomy, urinary diversion, ureterostomy, ileal conduit, patient-reported outcomes, patient-reported clinical outcomes, PRO scales, quality of life, social support, supportive care, physical, symptoms, complications, psychological, mental, emotions, etc. These terms are presented in Fig. 1 in the Supplementary Material Annex B.

**Figure 1:**
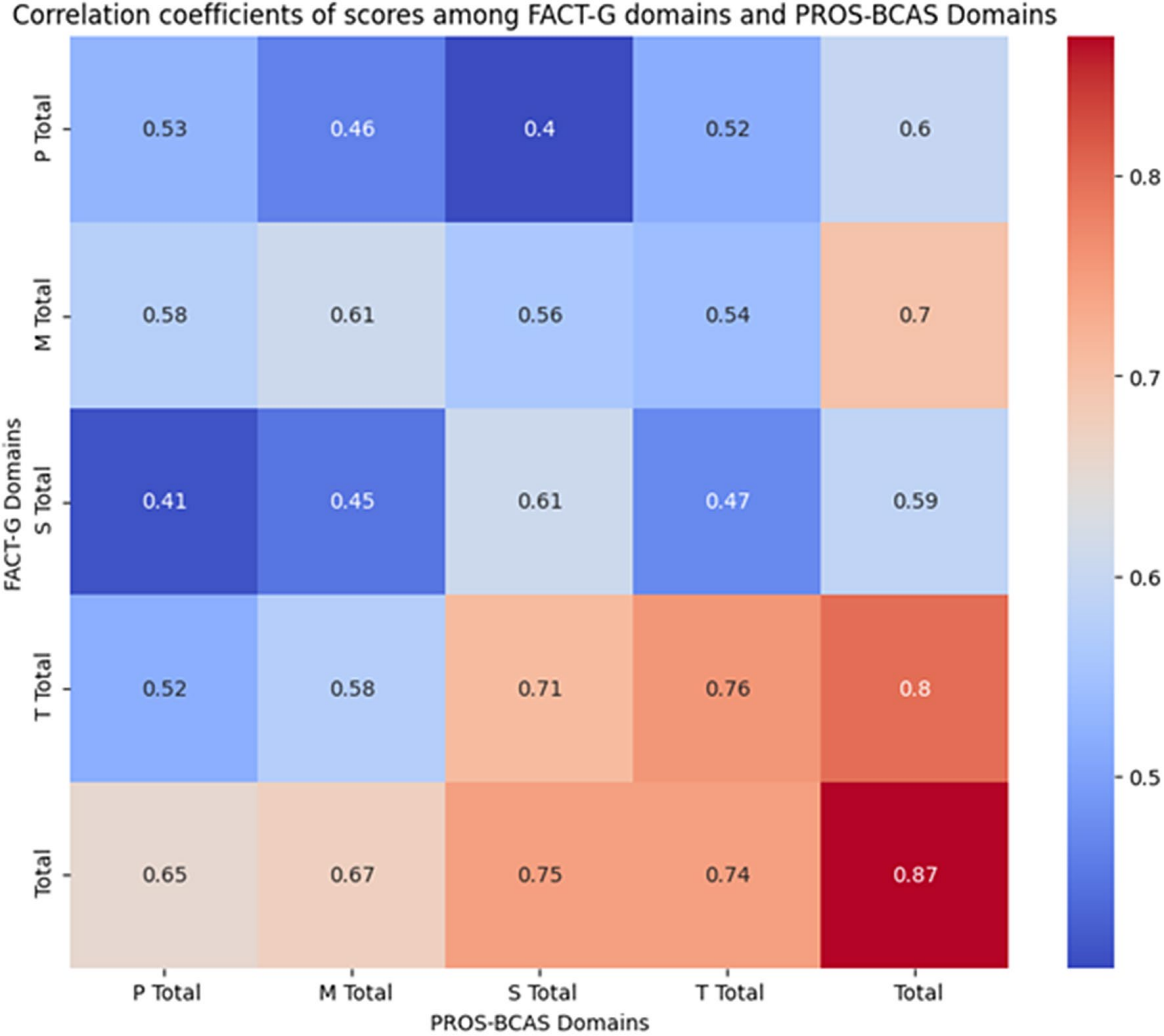
Heatmap Displaying Correlation among FACT-G Domains and PROS-BCAS Domains Scores. (*Note*: The deeper the color, the stronger the positive correlation).

We identified and summarized the 20 most relevant terms related to the quality of life of bladder cancer patients.Using Newman's systems model and Classical Test Theory (CTT) as the theoretical framework^34,35^, we determined 90 items through literature review, case review, and semi-structured interviews for the first round of expert consultation. These items were divided into four thematic domains: physiological domain (including specific symptom dimension (16 items), non-specific symptom dimension (9 items), daily living ability dimension (8 items)), psychological domain (including anxiety dimension (8 items), depression dimension (9 items), inferiority dimension (5 items)), social domain (including adaptation to stoma dimension (7 items), social activity dimension (4 items), social support dimension (7 items)), treatment domain (including compliance dimension (10 items), satisfaction dimension (7 items)).

### Delphi expert consultation

We invited 20 experts from five cities in China (Zhengzhou, Hangzhou, Shanghai, Kunming, and Nanchang) to participate in the consultation. The age range of these experts was 35–62 years, including 9 clinical doctors, 7 clinical nurses, and 4 university teachers. Among them, 5 experts held senior professional titles, and the remaining 15 experts held junior or lower professional titles. In terms of educational background, 4 experts held doctoral degrees, 4 held master's degrees, and the remaining 12 experts held bachelor's degrees.

We used the coefficient of variation (CV) and Kendall's W harmony coefficient to assess the consistency of expert opinions. We obtained a 100% questionnaire response rate in both rounds of expert consultation. The CV for the first and second rounds was 0.847 and 0.868, and Kendall's W was 0.245 and 0.219, respectively, indicating a high level of agreement among experts regarding the evaluation of each item.

Through two rounds of expert consultation, we eliminated, modified, and integrated the items and finally developed a preliminary scale for Patient-Reported Outcomes in Bladder Cancer with Abdominal Stoma (PROS-BCAS) consisting of four domains and 66 items.

### Pilot test

The pilot test was conducted using purposive sampling, and 20 bladder cancer patients with abdominal stoma were selected from a tertiary hospital in Nanchang, Jiangxi province, China, to evaluate the readability and usability of our initial scale. Based on patients' feedback, we made corresponding modifications to the questionnaire. During the survey, the purpose and content of the investigation were clearly explained to the patients, and the recall period was set as "the past two weeks." For patients who were unable to complete the questionnaire on their own, trained healthcare professionals assisted in filling it out. This post-operative collection of PROMs ensured that we accurately captured the patients' quality of life following their abdominal stoma creation. We used the Likert 5-point rating scale as the response options to accurately reflect the patients' actual conditions. To minimize potential biases introduced by researchers filling out the questionnaires, professional training on how to interact with participants in a non-leading manner was provided to all researchers involved in data collection. In addition, the process of filling out the questionnaires was recorded for future checks of potential biases.

### Reliability and validity evaluation

#### Participants

This study adopted a cross-sectional research design and included bladder cancer patients with abdominal stoma who received treatment in the stomal clinic of a tertiary hospital in Nanchang, China, between January 2020 and February 2023, using convenience sampling. All included patients had undergone open surgery for bladder cancer, followed by abdominal stoma creation. The inclusion criteria were: (1) Bladder cancer patients who have undergone radical cystectomy with cutaneous ureterostomy or ileal conduit; (2) aged 18 years and above, with effective language communication and comprehension ability; (3) no other severe physical or mental illnesses; (4) willing to participate in this study. The exclusion criteria were: (1) Patients with resistance or non-cooperation to the questionnaire survey; (2) Participants who withdrew midway; (3) Patients with other simultaneous cancers. Written informed consent was obtained from all participants before their participation in the study. We strictly adhered to all relevant data privacy and confidentiality ethical guidelines. To protect participants' privacy, all collected data were encrypted and participants' information was anonymized. This study aims to evaluate the reliability and validity of the patient-reported outcomes in bladder cancer with abdominal stoma according to the COSMIN reporting standard and has obtained approval from the Ethics Committee of the First Affiliated Hospital of Nanchang University.

### Research tools

The following tools were designed for effective data collection and analysis in this study: (1) General information survey: Developed by the research team to collect participants' basic demographic information and clinical data. (2) Self-report preliminary outcome scale for bladder cancer patients with abdominal wall stomas: This scale consists of 66 items used to evaluate self-reported outcomes in bladder cancer patients with abdominal wall stomas. (3) Functional Assessment of Cancer Therapy-Generic Scale (FACT-G)^36^: This scale consists of 27 items, covering dimensions of physical well-being, social/family well-being, emotional well-being, and functional well-being, for assessing the quality of life in cancer patients. The Cronbach's α coefficients for all dimensions of FACT-G were greater than 0.8, indicating good reliability and validity. All items in the above scales were rated on a Likert 5-point scale (range 0–4). Higher scores indicate better quality of life.

### Data collection and sample size calculation

The scales used in this study consisted of 66 items. According to the clinical epidemiological survey method, the preliminary sample size is usually set to 5 times the number of scale items, considering a 10% loss to follow-up or inefficiency rate^37^. Therefore, the minimum sample size required was calculated as 66 × 5 × (100% + 10%) = 363 cases. To ensure the robustness of the study results, a total of 398 questionnaires were distributed. Two tests were conducted. In the first round, 398 questionnaires were distributed, and 382 valid responses were collected, which were used for item analysis and exploratory factor analysis (response rate: 96.0%). The second test was conducted on 30 randomly selected patients (selected from the 382 patients in the first round) to evaluate the test–retest reliability after two weeks. Considering that bladder cancer patients with abdominal wall stomas are mostly elderly, to alleviate their fatigue and boredom, we used the Likert 5-point scale as the alternative answers. This rating scale scores from 0 to 4 based on the level of severity and can fully reflect the patients' symptoms, psychological status, social support, and treatment satisfaction.

### Reliability evaluation

We used test–retest reliability, split-half reliability, and Cronbach's α coefficient to assess the reliability. (1) Test–retest reliability: Pearson correlation coefficient was calculated, and a correlation coefficient > 0.75 indicates good test–retest reliability. To further evaluate the measurement error and stability of the scale, a two-way random model was chosen to calculate the intra-class correlation coefficient (ICC), standard measurement error (SEM), and smallest detectable change (SDC) for each domain. ICC > 0.75 indicates a high degree of consistency; smaller SEM indicates less measurement error and greater stability of measurement; smaller SDC indicates higher sensitivity of the scale. (2) Split-half reliability: Pearson correlation coefficient was calculated based on the scores obtained by participants in the two halves of the items. Split-half reliability is typically required to be ≥ 0.700. (3) Cronbach's α coefficient was calculated for the entire scale and each dimension to assess internal consistency. Generally, Cronbach's α coefficient ≥ 0.700 is considered acceptable.

### Validity assessment

We evaluated the content validity, structural validity, and criterion-related validity of the scale. We invited 6 experts to evaluate content validity. The inclusion criteria for experts were as follows: (1) at least 10 years of clinical medical or nursing experience; (2) bachelor's degree or higher; (3) associate senior or higher professional title; (4) main work and research areas include urology, ostomy care, nursing management, nursing education, etc.; (5) voluntary participation in this study. We calculated item-level content validity index (I-CVI) and scale-level content validity index (S-CVI) based on the experts' ratings. I-CVI is the ratio of the number of experts giving a rating of 3 or 4 to the total number of experts. S-CVI/Ave is the average of all item-level I-CVIs. Generally, I-CVI ≥ 0.780 and S-CVI/Ave ≥ 0.900 are considered acceptable.

We used exploratory factor analysis (EFA) to verify the structure of the scale. Before the analysis, we conducted the Kaiser–Meyer–Olkin (KMO) test to assess the appropriateness of the collected data for factor analysis. A larger KMO value indicates that the data is more suitable for factor analysis. KMO < 0.5 indicates that the data is not suitable for factor analysis, and KMO > 0.9 indicates that the data is highly suitable for factor analysis. The ideal factor loading coefficient should be > 0.70, but > 0.40 is also acceptable. When the above criteria are met, it indicates that the scale has a single dimension with sufficient structural validity. We selected the Functional Assessment of Cancer Therapy-General Scale (FACT-G) as the reference standard for criterion-related validity to evaluate the criterion-related validity of the PROS-BCAS scale. We calculated the Pearson correlation coefficients between the domain scores of FACT-G and the domain scores of PROS-BCAS to assess the correlation between the scale scores and widely accepted relevant concepts.

### Feasibility assessment

Feasibility assessment includes acceptance rate, completion rate, and completion time. Acceptance rate refers to the proportion of valid questionnaires received to the total number of questionnaires distributed. Completion rate refers to the proportion of valid questionnaires received to all questionnaires returned. Generally, the response rate and completion rate of a scale should exceed 85%. Scale completion time: It is generally considered acceptable if a scale can be completed within 20 min. If the completion time is too long, it may affect the authenticity and accuracy of the study.

### Statistical analysis

The statistical analysis was conducted by dedicated individuals within the research team who entered all the data into Excel using a parallel double data entry method. Cross-checks were performed to ensure data accuracy, and another specialist reviewed the data for further confirmation of its correctness. Statistical analysis and graphic generation were carried out using IBM SPSS Statistics 27.0 and Python language libraries such as NumPy, Matplotlib, NetworkX, and Seaborn. Missing data was handled using the nearest neighbor imputation method to fill in the gaps. Frequency and percentage were used to represent categorical data, while mean ± standard deviation was used for continuous data. We employed critical value comparison, correlation coefficient method, and homogeneity test for item selection in the scale. Reliability testing of the scale was performed using test–retest reliability, Cronbach's α coefficient, and split-half reliability. Content validity, structural validity, and criterion-related validity were utilized to assess the scale's validity.

### Ethics approval and consent to participate

This study was approved by the Ethics Committee of the First Affiliated Hospital of Nanchang University, in accordance with the principles of the Helsinki Declaration and its subsequent amendments or similar ethical standards. In addition, we followed the reporting guidelines of COSMIN (Consensus-based Standards for the selection of health status Measurement INstruments) in the design and execution of scale assessments. All participants in this study provided informed consent.

## Results

### Participant demographics

A total of 382 questionnaires were collected, with an average age of 68.04 ± 8.27 years. The acceptance rate of the scale was 96%, and the completion rate was 100%. The average completion time for the scale was 13.54 ± 2.09 min. Both the response rate and completion time of the scale were satisfactory, with rates exceeding 80% and completion within 20 min. Table 1 provides detailed information on the demographic and clinical characteristics of the bladder cancer patients with abdominal wall ostomy.

**Table 1:** Demographic and clinical characteristics of the study population (n, %).

Variables	Categories	Pre-survey sample (n = 20)	Formal survey sample (n = 382)	Test–retest reliability sample (n = 30)
Age (years)	< 60	2 (10.00)	48 (12.57)	2 (6.7)
	60–69	5 (25.00)	172 (45.03)	7 (23.3)
	70–79	8 (40.00)	135 (35.34)	14 (46.7)
	≥ 80	5 (25.00)	27 (7.07)	7 (23.3)
Gender	Male	14 (70.00)	330 (86.39)	25 (83.3)
	Female	6 (30.00)	52 (13.61)	5 (16.7)
Educational level	Primary school or below	8 (40.00)	276 (72.25)	19 (63.3)
	Junior high school	2 (10.00)	56 (14.66)	5 (16.7)
	Secondary vocational school/high school	6 (30.00)	28 (7.33)	4 (13.3)
	Junior college	2 (10.00)	17 (4.45)	1 (3.3)
	bachelor's degree or Above	2 (10.00)	5 (1.31)	1 (3.3)
Marital status	Married	16 (80.00)	359 (93.98)	27 (90.0)
	Unmarried	0 (0.00)	1 (0.26)	0 (0.0)
	Divorced	1 (5.00)	4 (1.05)	1 (3.3)
	Widowed	3 (15.00)	18 (4.71)	2 (6.7)
Place of residence	Rural	14 (70.00)	205 (53.92)	24 (80.0)
	Urban	6 (30.00)	177 (46.28)	6 (20.0)
Average monthly Income per Household	< 1200	2 (10.00)	34 (8.90)	1 (3.3)
	1200–2399	12 (60.00)	194 (50.79)	11 (36.7)
	2400–3199	4 (20.00)	117 (30.63)	15 (50.0)
	≥ 3200	2 (10.00)	37 (9.69)	3 (10.0)
Healthcare cost Payment method	Medical insurance	19 (95.00)	361 (94.51)	28 (93.3)
	Out-of-pocket	1 (5.00)	21 (5.50)	2 (6.7)
Type of stoma	Ileocecal bladder stoma	2 (10.00)	21 (5.50)	2 (6.7)
	Unilateral single ureteral skin Stoma	3 (15.00)	42 (11.00)	20 (66.7)
	Unilateral double ureteral skin stoma	6 (30.00)	87 (22.77)	5 (16.7)
	Bilateral ureteral skin stoma	9 (45.00)	232 (60.73)	3 (10.0)

### Item analysis

In the item analysis of the PROS-BCAS tool, a total of 66 items were analyzed. Based on the set criteria (i.e., CR value ≥ 3.000, item-total correlation ≥ 0.400, corrected item-total correlation (CITC) ≥ 0.400, Cronbach's Alpha after deleting the item ≤ 0.929, communality ≥ 0.200, factor loading ≥ 0.400), items that did not meet the selection criteria and had ≥ 4 indicator items were deleted. We deleted 22 items and retained 44 items for further exploratory factor analysis. The results of the item analysis are shown in Table 1 in the Supplementary Material Annex B.

### Validity testing

#### Content validity

In this evaluation, 6 experts assessed the content validity. The results showed a content validity index (S-CVI/Ave) of 0.992 for the scale and an item content validity index (I-CVI) ranging from 0.83 to 1.

### Structural validity

We conducted exploratory factor analysis on the 44 items in the physiological, psychological, social, and treatment domains. The Kaiser–Meyer–Olkin (KMO) test values for each domain were 0.847, 0.760, 0.843, and 0.904, all of which exceeded the threshold of 0.6, indicating that the data were suitable for factor analysis. Additionally, the Bartlett's test of sphericity for each domain yielded statistically significant results (*χ*^*2*^ = 3284.652, *p* < 0.001;* χ*^*2*^ = 1607.623, *p* < 0.001; *χ*^*2*^ = 2705.276,* p* < 0.001; *χ*^*2*^ = 8197.548, *p* < 0.001), further supporting the decision to conduct factor analysis. We used principal component analysis and performed an orthogonal rotation with varimax rotation. The results showed that each domain could be represented by two common factors, with cumulative variance contribution rates of 68.492%, 60.897%, 73.621%, and 77.818% for the respective domains. These results exceed the acceptance criterion of 40%, indicating that these common factors effectively explain the variability in each domain. Thus, we ultimately constructed a scale consisting of 8 dimensions and 44 items. We conducted exploratory factor analysis on the 44 items in the physiological, psychological, social, and treatment domains. The Kaiser–Meyer–Olkin (KMO) test values for each domain were 0.847, 0.760, 0.843, and 0.904, all of which exceeded the threshold of 0.6, indicating that the data were suitable for factor analysis. Additionally, the Bartlett's test of sphericity for each domain yielded statistically significant results (χ2 = 3284.652, *p* < 0.001; χ2 = 1607.623, *p* < 0.001; χ2 = 2705.276, *p* < 0.001; χ2 = 8197.548, *p* < 0.001), further supporting the decision to conduct factor analysis. We used principal component analysis and performed an orthogonal rotation with varimax rotation. The results showed that each domain could be represented by two common factors, with cumulative variance contribution rates of 68.492%, 60.897%, 73.621%, and 77.818% for the respective domains. These results exceed the acceptance criterion of 40%, indicating that these common factors effectively explain the variability in each domain. Thus, we ultimately constructed a scale consisting of 8 dimensions and 44 items. The relevant factor analysis path diagram is presented in Fig. 2 in the Supplementary Material Annex B.

**Figure 2:**
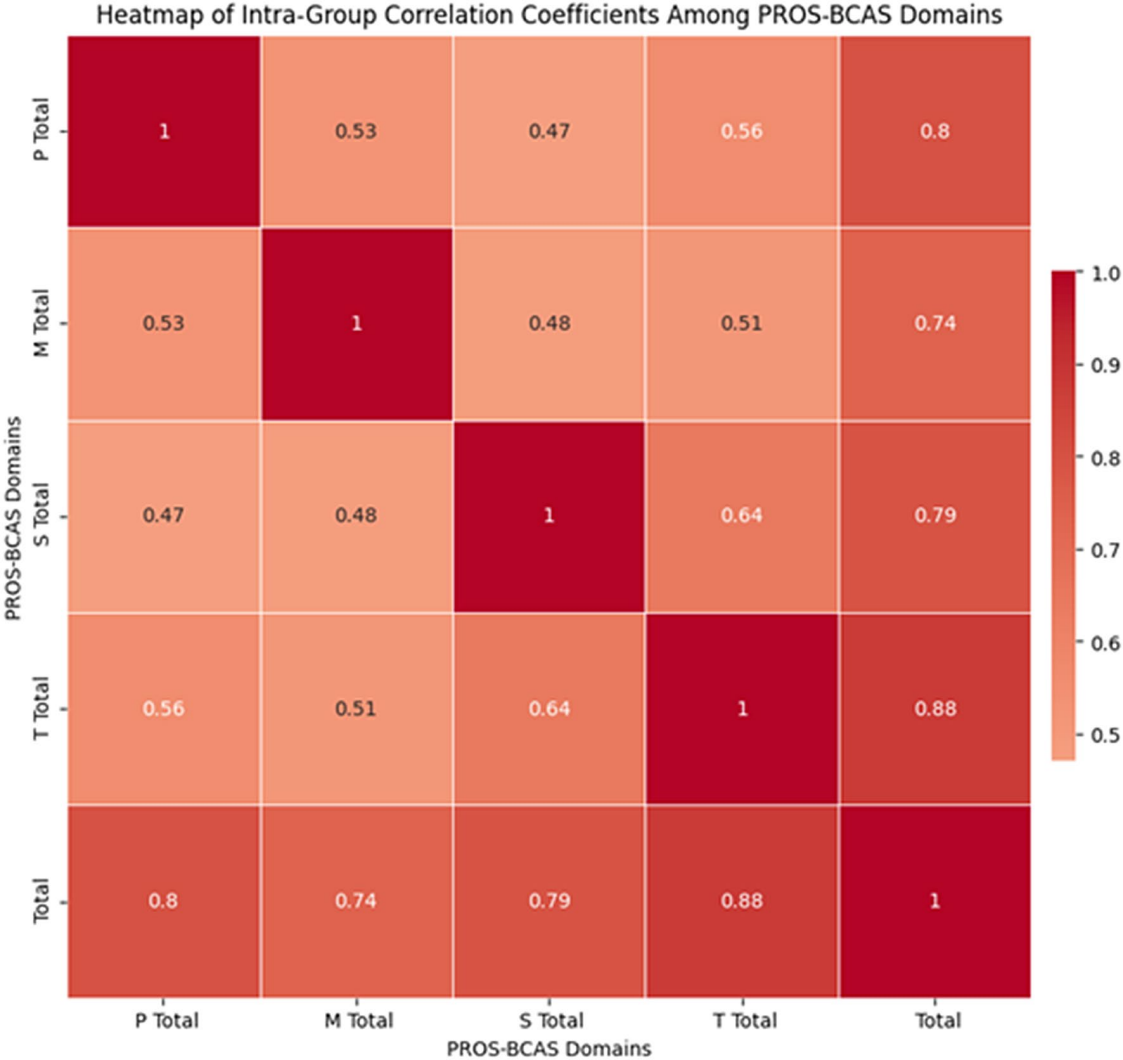
Heatmap Showing Correlation Strength among PROS-BCAS Domains Scores. (*Note*: The deeper the color, the stronger the positive correlation).

### Convergent validity

We used Pearson correlation coefficients to demonstrate the relationship between the domain scores of the FACT-G and PROS-BCAS. We visualized this relationship with a heatmap, where the color intensity represents the strength of the correlation. All domain correlations reached a significant level (*p* < 0.01), with Pearson correlation coefficients ranging from 0.404 to 0.870. All values exceeded 0.4, indicating a moderate to strong correlation. These results further validate the convergent validity of the PROS-BCAS scale. Refer to Table 2 in the Supplementary Material Annex B and Fig. 1 for more details.

### Reliability evaluation

#### Internal consistency

We used Pearson correlation coefficients and a heatmap to demonstrate the correlation between domains. In the heatmap, the color intensity represents the magnitude of the correlation, with lighter colors indicating lower correlation and darker colors indicating higher correlation. The Pearson correlation coefficients between the domain scores and the overall score ranged from 0.739 to 0.880, showing significant correlations (*p* < 0.01). Similarly, significant correlations were observed between domains, with coefficients ranging from 0.471 to 0.643 (*p* < 0.01), all exceeding 0.4. These results further support the consistency and overall reliability of the scale and its domains. See Table 3Supplementary Material Annex B and Fig. 2 for more details.

### Test–retest reliability

We evaluated the reliability and stability of the scale. The Cronbach's alpha coefficient for the overall scale was 0.954. This, along with the split-half reliability of 0.977 and the test–retest reliability of 0.927, indicates good reliability and stability. The Cronbach's alpha coefficients, split-half reliabilities, and test–retest reliabilities of the domains also confirmed their reliability and stability, as shown in Table 4 in the Supplementary Material Annex B. The intraclass correlation coefficient (ICC) for the scale was 0.954, with domain values ranging from 0.860 to 0.972, demonstrating high consistency across different time points. The standard error of measurement (SEM) for the scale was 5.32, and the smallest detectable change (SDC) was 14.747. The SEM and SDC of each domain also indicate the stability and reliability of the scale.

## Discussion

The aim of this study was to develop and validate a scale specifically designed for Chinese bladder cancer patients with an abdominal stoma to report outcomes, called the Patient-Reported Outcomes Scale for Bladder Cancer with Abdominal Stoma (PROS-BCAS). The PROS-BCAS scale developed in this study provides a valuable tool for comprehensively evaluating the health-related quality of life of bladder cancer patients with an abdominal stoma. It covers key aspects including physiological, psychological, social, and treatment domains, accurately reflecting patients' health status and experiences.

In the initial design phase, we constructed a scale framework consisting of four domains and eleven dimensions, with a total of 66 items. However, after item analysis, we found that some dimensions did not meet the predetermined retention criteria. These items were excluded because they were either not expressed accurately or were difficult for patients to understand and answer. Although these items may have some value in certain aspects, retaining them could have a negative impact on the overall reliability and validity of the scale. After careful consideration, we optimized our scale to encompass four domains and eight dimensions with 44 items, effectively covering physiological, psychological, social, and treatment aspects. This streamlined version maintains excellent reliability and validity, effectively capturing the diverse impacts on bladder cancer patients with abdominal stomas.

The content validity of the scale was highly recognized by experts, with high S-CVI and I-CVI, indicating that our constructed scale had logical and consistent structure and content. Exploratory factor analysis revealed good structural validity of the scale. Our data were suitable for factor analysis, and all domains could be represented by two common factors, which effectively explained the differences in each domain. This further demonstrated the advantage of our scale in terms of structural validity.

Our comparative analysis with the FACT-G scale revealed significant correlations across all domains, affirming the criterion-related validity of the PROS-BCAS scale. This correlation underscores the accuracy of our scale in assessing the specific outcomes of bladder cancer patients with abdominal stomas. While both scales share conceptual similarities and cover comparable domains, the PROS-BCAS scale is distinct in its tailored approach to the unique experiences of our target population. Unlike FACT-G, which is general in scope, the PROS-BCAS scale delves into specifics pertinent to bladder cancer patients with stomas, addressing nuanced aspects of their condition. This targeted approach potentially leads to variations in patient responses, highlighting the necessity and relevance of our scale in capturing the detailed health-related quality of life impacts in this particular group. Our findings thus not only validate the effectiveness of the PROS-BCAS scale but also emphasize its unique contribution in the realm of bladder cancer patient assessments.

The reliability evaluation of the scale demonstrates significant internal consistency both within individual domains and overall, indicating a strong relationship among the different domains of the scale. This is crucial for ensuring the reliability of the scale. Belita et al.'s study suggests that a highly effective and reliable scale should have a Cronbach's α coefficient and split-half reliability above 0.7^38^. In our research, the PROS-BCAS scale exhibited excellent internal consistency, with a Cronbach's α coefficient of 0.954 and a split-half reliability of 0.977. These results meet Belita et al.'s criteria, which is crucial for ensuring the scale's ongoing use.

The development and validation of the PROS-BCAS scale provide clinical medical staff with an evaluative tool of significant utility in practical applications. It enables a comprehensive understanding and assessment of patients' complex needs post-abdominal stoma creation, encompassing physiological, psychological, social, and treatment aspects. Furthermore, this tool plays a crucial role in guiding the adjustment and improvement of patient management strategies, optimizing treatment methods, enhancing service quality, and increasing patient satisfaction. In the ever-evolving medical field, the PROS-BCAS scale, as an effective assessment tool, offers more personalized and integrated care plans for bladder cancer patients.

### Clinical significance and limitations

This study successfully developed and validated a self-assessment scale for patients with bladder cancer who underwent abdominal wall creation of a stoma, called the Patient-Reported Outcome Scale-Bladder Cancer Abdominal Stoma (PROS-BCAS). The scale comprehensively evaluates the patients' physiological, psychological, social, and treatment conditions, accurately reflecting their health and quality of life. It can serve as a reference for personalized treatment plans and is an important tool for assessing different treatment methods and service quality, ultimately improving patient satisfaction. However, there are some limitations in this study, such as the potential bias introduced by self-assessment, the absence of confirmatory factor analysis and assess of responsiveness. Additionally, this scale was specifically designed for Chinese patients and its cross-cultural validation is warranted in future studies. Despite these limitations, we believe that with further revision and validation, this scale will become a valuable tool for assessing the health outcomes of patients with bladder cancer who underwent abdominal wall creation of a stoma. We encourage researchers to test this scale in different populations to enhance its widespread applicability. Overall, this study successfully developed and preliminarily validated the PROS-BCAS scale, providing a new tool for evaluating the health status of patients with bladder cancer who underwent abdominal wall creation of a stoma, and thus having significant clinical and academic value.

## Conclusion

In this study, we successfully developed and validated a Patient-Reported Outcome Measurements (PROMs) scale, PROS-BCAS, specifically for Chinese patients with bladder cancer abdominal wall stoma. This scale comprehensively covers four key domains: physiological, psychological, social, and treatment; providing patients with a systematic and comprehensive tool for assessing their health and quality of life. Our research results demonstrate that this scale exhibits good performance in terms of structural validity, content validity, and internal consistency, and possesses strong application potential. However, we must acknowledge the limitations of this study, including potential subjectivity bias, potential unmeasured factors, and uncertainty regarding its applicability to other cultural settings. Future research should further test and validate the scale across different cultures and assess its validity to better serve the health assessment and treatment of patients with bladder cancer abdominal wall stoma (Supplementary Material Annex B).

### Supplementary Information

Supplementary Information 1.

Supplementary Information 2.

## Data Availability

The datasets used and/or analysed during the current study are available from the corresponding author on reasonable request.
