# Supplementary material for: Development and preliminary validation of a PROS scale for Chinese bladder cancer patients with abdominal stoma
Source: Sci Rep. 2024 Jan 25;14:2187. doi: 10.1038/s41598-024-52624-0 (PMC10810889; doi:10.1038/s41598-024-52624-0)
Supplement: Supplementary file 1 — Supplementary Information 1. [file 41598_2024_52624_MOESM1_ESM.docx]

| **Supplementary Material Annex A.**The PROS Scale for Chinese Bladder Cancer Patients with Abdominal Stoma | | | | | |
| --- | --- | --- | --- | --- | --- |
| Items | Scoring Description | | | | |
| **Physiological Domain** | | | | | |
| **Specific Symptoms** | None | Mild | Present | Moderate to severe | Very severe |
| * Have you noticed any abnormal changes in the color of your stoma? | 4 | 3 | 2 | 1 | 0 |
| * Have you recently experienced pain in your stoma? | 4 | 3 | 2 | 1 | 0 |
| * Have you recently faced difficulties in urinating? | 4 | 3 | 2 | 1 | 0 |
| * Is the color of your urine yellowish? | 4 | 3 | 2 | 1 | 0 |
| * Have you recently noticed blood in your urine? | 4 | 3 | 2 | 1 | 0 |
| * Does your urine appear cloudy? | 4 | 3 | 2 | 1 | 0 |
| **Non-specific Symptoms** | None | Slight | Present | Significant | Very significant |
| * How has your recent sleep been? Do you find it difficult to fall asleep or wake up easily during sleep? | 4 | 3 | 2 | 1 | 0 |
| * Do you feel that your appetite is not as good as before? | 4 | 3 | 2 | 1 | 0 |
| * Do you experience abdominal bloating or discomfort? | 4 | 3 | 2 | 1 | 0 |
| * Do you feel pain in your lower back? | 4 | 3 | 2 | 1 | 0 |
| * Do you notice swelling in your lower limbs? | 4 | 3 | 2 | 1 | 0 |
| * Have you experienced a recent weight loss? | 4 | 3 | 2 | 1 | 0 |
| **Psychological Domain** | | | | | |
| **Anxiety** | Not worried | Slightly worried | Moderate | Somewhat worried | Very worried |
| * When the ostomy bag has more urine, do you worry about leaks leading to embarrassment? | 4 | 3 | 2 | 1 | 0 |
| * Are you concerned about potential ostomy issues or recurrence of cancer? | 4 | 3 | 2 | 1 | 0 |
| * Are you worried about body odor? | 4 | 3 | 2 | 1 | 0 |
| * Do you feel unexplained fear? | 4 | 3 | 2 | 1 | 0 |
| * Facing the stoma, are you worried about your health condition? | 4 | 3 | 2 | 1 | 0 |
| **Inferiority** | Never | Occasionally | Sometimes | Frequently | Always |
| * Do you feel annoyed or troubled by the stoma? | 4 | 3 | 2 | 1 | 0 |
| Are you satisfied with your current body image? | 4 | 3 | 2 | 1 | 0 |
| * Do you believe you have become dependent on others for care because of the stoma? | 4 | 3 | 2 | 1 | 0 |
| * Do you think the stoma has made you lose hope and confidence in life? | 4 | 3 | 2 | 1 | 0 |
| **Social Domain** | | | | | |
| **Stoma Indication** | No impact | Slight impact | Light | Moderate | Very significant impact |
| * Has the ostomy affected your daily activities, such as dressing, bathing, and sleeping? | 4 | 3 | 2 | 1 | 0 |
| * Has the ostomy influenced your role and status within your family or at work? | 4 | 3 | 2 | 1 | 0 |
| * Do you feel embarrassed in public places because of the ostomy? | 4 | 3 | 2 | 1 | 0 |
| * When interacting with others, do you feel isolated due to the ostomy? | 4 | 3 | 2 | 1 | 0 |
| * Do you believe you are more prone to getting sick because of the ostomy? | 4 | 3 | 2 | 1 | 0 |
| **Social Support** | Never | Occasionally | Sometimes | Frequently | Always |
| Do you feel support and care from your family? | 4 | 3 | 2 | 1 | 0 |
| Can you continue your hobbies and interests? | 4 | 3 | 2 | 1 | 0 |
| Do you see the ostomy as a part of your body? | 4 | 3 | 2 | 1 | 0 |
| Do your family and friends help you understand and manage the ostomy? | 4 | 3 | 2 | 1 | 0 |
| **Treatment Domain** | | | | | |
| **Compliance** | Unable to do at all | Somewhat difficult | Able to do | Mostly able to do | Able to do very easily and well |
| * Do you find changing the urostomy bag independently challenging? | 4 | 3 | 2 | 1 | 0 |
| Do you maintain cleanliness daily and change the ostomy bag promptly after showering? | 4 | 3 | 2 | 1 | 0 |
| Do you ensure a balanced diet and consume 1500-2000ml of water daily? | 4 | 3 | 2 | 1 | 0 |
| * Do you avoid rigorous exercises or activities that increase abdominal pressure? | 4 | 3 | 2 | 1 | 0 |
| Do you change the urostomy bag at the recommended frequency and immediately if there's leakage? | 4 | 3 | 2 | 1 | 0 |
| * Do you avoid using alcohol and iodine when changing the ostomy? | 4 | 3 | 2 | 1 | 0 |
| Do you regularly visit the hospital for check-ups and to change related accessories? | 4 | 3 | 2 | 1 | 0 |
| **Satisfaction** | Dissatisfied | Somewhat dissatisfied | Satisfied | Very satisfied | Extremely satisfied |
| Do hospitals in your region provide you with convenient and professional ostomy care? | 4 | 3 | 2 | 1 | 0 |
| Have you received adequate knowledge and skills for ostomy care? | 4 | 3 | 2 | 1 | 0 |
| Do you find the current treatment method beneficial? | 4 | 3 | 2 | 1 | 0 |
| Are you satisfied with the service attitude of the doctors and nurses? | 4 | 3 | 2 | 1 | 0 |
| Do you feel your family provides sufficient support? | 4 | 3 | 2 | 1 | 0 |
| Do you think the ostomy care costs are reasonable? | 4 | 3 | 2 | 1 | 0 |
| Are you optimistic about future treatments and care? | 4 | 3 | 2 | 1 | 0 |
| Note: Each item is scored on a scale of 0-4.The total score of the scale is 176, with higher scores indicating a better quality of life. Questions marked with "*" should be reverse scored during scoring. | | | | | |
