# Supplementary material for: Development and preliminary validation of a PROS scale for Chinese bladder cancer patients with abdominal stoma
Source: Sci Rep. 2024 Jan 25;14:2187. doi: 10.1038/s41598-024-52624-0 (PMC10810889; doi:10.1038/s41598-024-52624-0)
Supplement: Supplementary file 2 — Supplementary Information 2. [file 41598_2024_52624_MOESM2_ESM.docx]

**Supplementary Material Annex B**

*Supplementary Figure. 1:Top 20 Terms Related to the Quality of Life of Bladder Cancer Patients with Abdominal Ostomies*


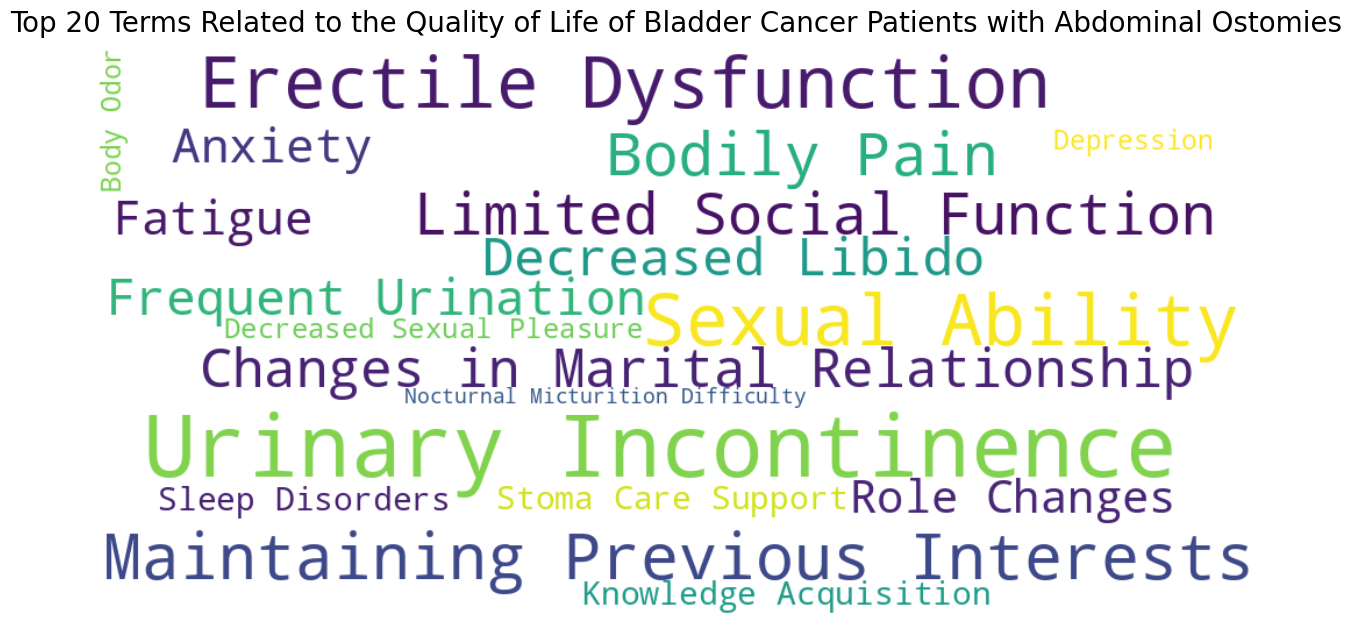


*Supplementary Figure. 2:Factor Analysis Path Diagram.*


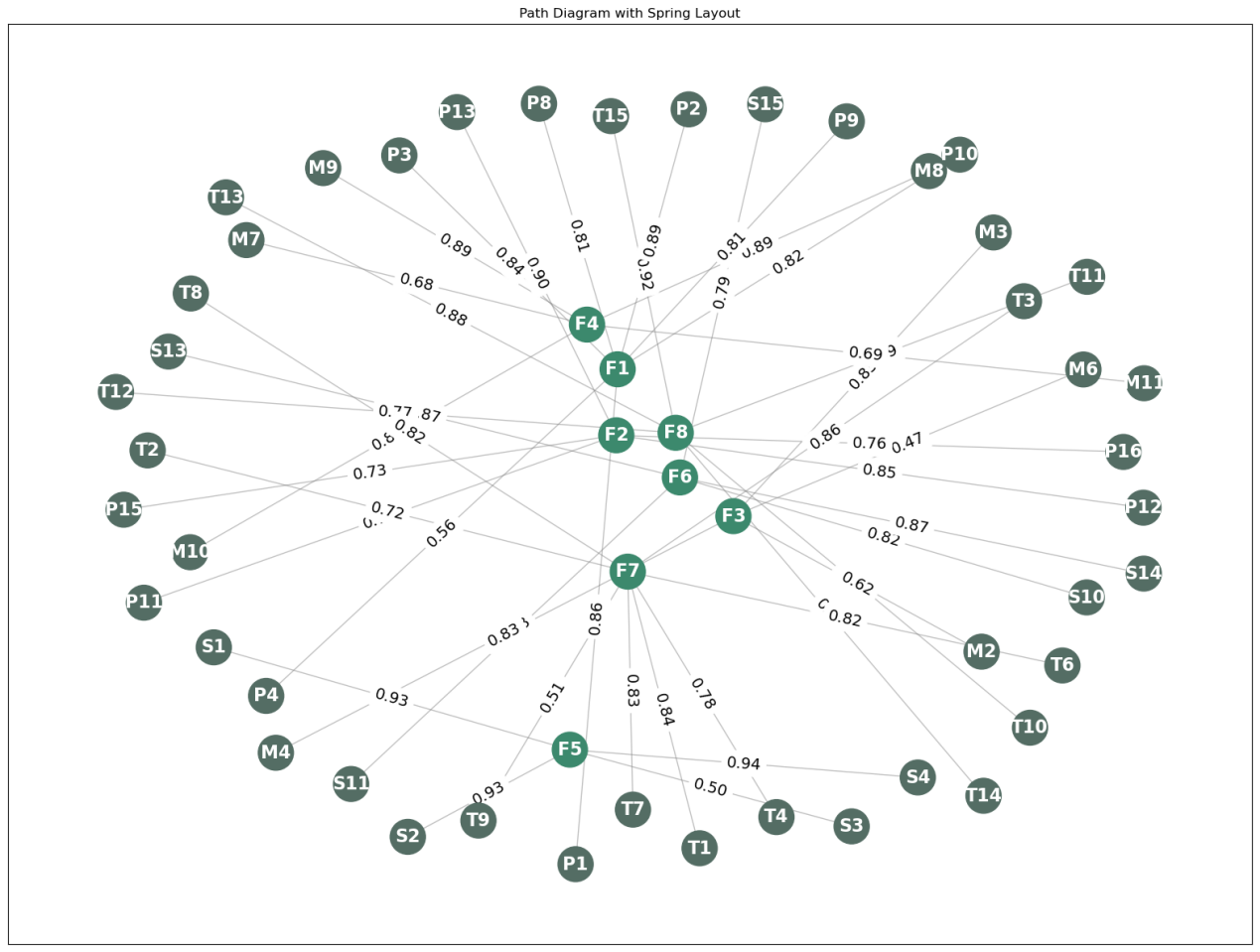


Note: In the path diagram, the eight dimensions are represented by F1 to F8, specifically: F1 represents the 'Specific Symptoms' dimension, F2 represents the 'Non-specific Symptoms' dimension, F3 represents the 'Anxiety' dimension, F4 represents the 'Inferiority' dimension, F5 represents the 'Stoma Indication' dimension, F6 represents the 'Social Support' dimension, F7 represents the 'Compliance' dimension, and F8 represents the 'Satisfaction' dimension.

| *Supplementary Table 1:* Item analysis of the PROS-BCAS tool (n=382) | | | | | | | | |
| --- | --- | --- | --- | --- | --- | --- | --- | --- |
| Item | Critical Ratio | Correlation coefficient | | Homogeneity Test | | | Number of substandard indicators | Comment |
|  | CR value | Item-Total Correlation | Corrected Item-Total Correlation(CITC) | Cronbach's Alpha  if Item Deleted | Communalities | Factor loading |  |  |
| P1 | 10.132*** | .514** | .487 | .928 | #.193 | .440 | 1 | Remain |
| P2 | 9.39*** | .475** | .448 | .928 | #.113 | #.337 | 2 | Remain |
| P3 | 11.799*** | .567** | .543 | .928 | .227 | .476 | 0 | Remain |
| P4 | 10.372*** | .487** | .462 | .928 | .201 | .449 | 0 | Remain |
| #P5 | 4.826*** | #.218** | #.186 | #.93 | #.01 | #.101 | 5 | Delete |
| #P6 | 5.935*** | #.280** | #.254 | .929 | #.055 | #.235 | 4 | Delete |
| #P7 | 6.324*** | #.375** | #.345 | .929 | #.154 | #.392 | 4 | Delete |
| P8 | 9.164*** | .532** | .507 | .928 | .258 | .508 | 0 | Remain |
| P9 | 10.506*** | .558** | .535 | .928 | .252 | .502 | 0 | Remain |
| P10 | 10.733*** | .548** | .523 | .928 | .213 | .461 | 0 | Remain |
| P11 | 10.878*** | .541** | .515 | .928 | .330 | .574 | 0 | Remain |
| P12 | 9.603*** | .483** | .453 | .928 | .326 | .571 | 0 | Remain |
| P13 | 11.54*** | .572** | .542 | .927 | .462 | .679 | 0 | Remain |
| #P14 | #2.451* | #.137** | #.092 | #.931 | #.001 | #-0.015 | 6 | Delete |
| P15 | 9.013*** | .532** | .504 | .928 | .357 | .598 | 0 | Remain |
| P16 | 12.668*** | .627** | .603 | .927 | .499 | .706 | 0 | Remain |
| #P17 | 3.501** | #.216** | #.178 | #.93 | #.058 | #.24 | 5 | Delete |
| #P18 | 7.681*** | .403** | #.371 | .929 | #.119 | #.344 | 3 | Delete |
| #P19 | 3.476** | #.173** | #.13 | #.93 | #.017 | #.131 | 5 | Delete |
| #M1 | #-0.228 | #-0.018 | #-0.055 | #.931 | #.004 | #-0.065 | 6 | Delete |
| M2 | 8.64*** | .524** | .496 | .928 | .350 | .592 | 0 | Remain |
| M3 | 9.131*** | .459** | .427 | .928 | #.149 | #.386 | 2 | Remain |
| M4 | 7.178*** | .438** | .405 | .928 | #.144 | #.38 | 2 | Remain |
| #M5 | 6.83*** | #.365** | #.33 | .929 | #.04 | #.201 | 4 | Delete |
| M6 | 10.068*** | .532** | .509 | .928 | .324 | .569 | 0 | Remain |
| M7 | 11.954*** | .512** | .483 | .928 | #.151 | #.388 | 2 | Remain |
| M8 | 9.396*** | .479** | .451 | .928 | #.152 | #.39 | 2 | Remain |
| M9 | 11.541*** | .515** | .488 | .928 | #.186 | .432 | 1 | Remain |
| M10 | 11.672*** | .536** | .510 | .928 | .219 | .468 | 0 | Remain |
| M11 | 8.981*** | .421** | #.39 | .928 | #.084 | #.29 | 3 | Remain |
| #M12 | 4.903*** | #.280** | #.241 | #.93 | #.015 | #.123 | 5 | Delete |
| #M13 | 6.239*** | #.265** | #.23 | .929 | #.015 | #.123 | 4 | Delete |
| #M14 | 5.244*** | #.219** | #.182 | #.93 | #.002 | #.043 | 5 | Delete |
| #M15 | 3.992*** | #.166** | #.125 | #.93 | #.003 | #-0.054 | 5 | Delete |
| #M16 | #2.922** | #.138** | #.099 | #.93 | #.001 | #-0.03 | 6 | Delete |
| #M17 | #.442 | #-0.001 | #-0.043 | #.932 | #.033 | #-0.183 | 6 | Delete |
| S1 | 12.243*** | .631** | .608 | .927 | .429 | .655 | 0 | Remain |
| S2 | 12.168*** | .606** | .582 | .927 | .390 | .624 | 0 | Remain |
| S3 | 7.711*** | .451** | .418 | .928 | #.156 | #.396 | 2 | Remain |
| S4 | 12.793*** | .621** | .598 | .927 | .415 | .644 | 0 | Remain |
| #S5 | #-0.783 | #-0.065 | #-0.103 | #.932 | #.013 | #-0.112 | 6 | Delete |
| #S6 | #.403 | #.003 | #-0.034 | #.931 | #.002 | #-0.05 | 6 | Delete |
| #S7 | #-1.019 | #.001 | #-0.029 | #.931 | #.00 | #-0.004 | 6 | Delete |
| #S8 | 3.505*** | #.232** | #.206 | .929 | #.038 | #0.196 | 4 | Delete |
| #S9 | #.820 | #-0.002 | #-0.033 | #.931 | #.004 | #-0.063 | 6 | Delete |
| S10 | 10.689*** | .555** | .530 | .928 | .359 | .599 | 0 | Remain |
| S11 | 8.203*** | .500** | .474 | .928 | .290 | .539 | 0 | Remain |
| #S12 | #1.178 | #.086 | #.059 | #.93 | #.003 | #.053 | 6 | Delete |
| S13 | 8.744*** | .524** | .497 | .928 | .321 | .566 | 0 | Remain |
| S14 | 8.358*** | .490** | .461 | .928 | .276 | .525 | 0 | Remain |
| S15 | 8.072*** | .496** | .470 | .928 | .290 | .538 | 0 | Remain |
| T1 | 11.908*** | .667** | .648 | .927 | .557 | .746 | 0 | Remain |
| T2 | 13.166*** | .621** | .601 | .927 | .466 | .682 | 0 | Remain |
| T3 | 13.3*** | .690** | .671 | .927 | .583 | .763 | 0 | Remain |
| T4 | 13.186*** | .693** | .676 | .927 | .544 | .737 | 0 | Remain |
| #T5 | 5.44*** | #.236** | #.212 | .929 | #.054 | #.233 | 4 | Delete |
| T6 | 16.042*** | .745** | .731 | .927 | .626 | .791 | 0 | Remain |
| T7 | 13.765*** | .725** | .723 | .927 | .648 | .805 | 0 | Remain |
| T8 | 8.618*** | .580** | .691 | .927 | .586 | .765 | 0 | Remain |
| T9 | 13.63*** | .708** | .556 | .927 | .494 | .703 | 0 | Remain |
| T10 | 13.317*** | .739** | .709 | .927 | .665 | .815 | 0 | Remain |
| T11 | 12.014*** | .666** | .646 | .927 | .587 | .766 | 0 | Remain |
| T12 | 10.241*** | .606** | .585 | .927 | .476 | .690 | 0 | Remain |
| T13 | 12.000*** | .659** | .639 | .927 | .573 | .757 | 0 | Remain |
| T14 | 6.261*** | .412** | #.382 | .929 | .301 | .549 | 1 | Remain |
| T15 | 6.360*** | .418** | #.388 | .928 | .306 | .554 | 1 | Remain |
| Criterion | ≥3.000 | ≥.400 | ≥.400 | ≤**.929** | ≥.200 | ≥.400 | / | / |

Note: *p < .05; **p < .01; ***p < .001.

# Denote the indicator does not meet the criteria

The value .929 in bold represents the internal consistency coefficient of the scale.

Abbreviation: CR, critical ratio

| *Supplementary Table 2:* Correlation coefficients of scores among FACT-G domains and PROS-BCAS Domains | | | | | |
| --- | --- | --- | --- | --- | --- |
| FACT-G Domains | PROS-BCAS Domains | | | | |
|  | PROS-BCAS P Total | PROS-BCAS M Total | PROS-BCAS S Total | PROS-BCAS T Total | PROS-BCAS Total |
| FACT-G P Total | .528** | .463** | .404** | .517** | .597** |
| FACT-G M Total | .577** | .608** | .561** | .543** | .698** |
| FACT-G S Total | .412** | .448** | .608** | .473** | .591** |
| FACT-G T Total | .520** | .576** | .708** | .756** | .799** |
| FACT-G Total | .654** | .673** | .746** | .745** | .870** |

Note: FACT-G and PROS-BCAS Domains include P (Physiological), M (Mental), S (Social), and T (Treatment). 'Total' refers to the total score of each scale. ** Correlation is significant at the 0.01 level (2-tailed).

*Supplementary Table 3:* Intra-Group Correlation Coefficients Among PROS-BCAS Domains Using Pearson's Method

| Domains | P Total | M Total | S Total | T Total | Total |
| --- | --- | --- | --- | --- | --- |
| P Total | 1 | .532** | .471** | .559** | .800** |
| M Total | .532** | 1 | .485** | .509** | .739** |
| S Total | .471** | .485** | 1.00 | .643** | .792** |
| T Total | .559** | .509** | .643** | 1 | .880** |
| Total | .800** | .739** | .792** | .880** | 1 |

Note: This table shows Pearson's correlation coefficients among the PROS-BCAS domains. The rows and columns represent the various domains, including Physiological (P Total), Mental (M Total), Social (S Total), Treatment (T Total), and the Total score (Total). '**' indicates a significant correlation at the 0.01 level.

| *Supplementary Table 4:* Comprehensive Evaluation of the Reliability of the PROS-BCAS | | | | | | | | |
| --- | --- | --- | --- | --- | --- | --- | --- | --- |
| Domains | Subdomains | Number of Items | Cronbach's Alpha Coefficient | Split-Half Reliability | Test–retest reliability | ICC（95%CI） | SEM | SDC |
|  |  |  |  |  |  |  |  |  |
| Physiological domains | Specific Symptoms | 6 | .889 | .935 | .787** | .861(.711-.933） | 2.719 | 7.537 |
|  | Non-specific Symptoms | 6 |  |  |  |  |  |  |
| Mental domains | Anxiety | 5 | .801 | .896 | .778** | .860(.712-.933) | 3.461 | 9.595 |
|  | Inferiority | 4 |  |  |  |  |  |  |
| Social domains | Stoma indication | 5 | .884 | .928 | .939** | .965(.926-.983) | 1.226 | 3.399 |
|  | Social support | 4 |  |  |  |  |  |  |
| Treatment domains | Compliance | 7 | .954 | .973 | .956** | .972(.942-.987) | 1.231 | 3.412 |
|  | Satisfaction | 7 |  |  |  |  |  |  |
| Total scale | —— | 44 | .954 | .977 | .927** | .954(.904-.978) | 5.32 | 14.747 |

Note: The test-retest reliability is calculated based on a sample of 30 participants. "Domain" refers to the main field, while "Subdomains" represent various dimensions within that field. ICC (Intraclass Correlation Coefficient) indicates the consistency of scores, SEM (Standard Error of Measurement) measures individual scores or measurement accuracy, and SDC (Smallest Detectable Change) represents the minimum change in scores that can be attributed to true change beyond measurement error. The formula for SEM is as follows:
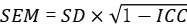
.The formula for SDC is as follows:
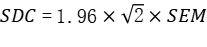
. '**' indicates significance at the 0.01 level.
